# Cold-sensing regulates *Drosophila* growth through insulin-producing cells

**DOI:** 10.1038/ncomms10083

**Published:** 2015-12-09

**Authors:** Qiaoran Li, Zhefeng Gong

**Affiliations:** 1 Department of Neurobiology, Key Laboratory of Medical Neurobiology of the Ministry of Health of China, Key Laboratory of Neurobiology, Zhejiang University School of Medicine, Hangzhou, Zhejiang 310058, China

## Abstract

Across phyla, body size is linked to climate. For example, rearing fruit flies at lower temperatures results in bigger body sizes than those observed at higher temperatures. The underlying molecular basis of this effect is poorly understood. Here we provide evidence that the temperature-dependent regulation of *Drosophila* body size depends on a group of cold-sensing neurons and insulin-producing cells (IPCs). Electrically silencing IPCs completely abolishes the body size increase induced by cold temperature. IPCs are directly innervated by cold-sensing neurons. Stimulation of these cold-sensing neurons activates IPCs, promotes synthesis and secretion of *Drosophila* insulin-like peptides and induces a larger body size, mimicking the effects of rearing the flies in cold temperature. Taken together, these findings reveal a neuronal circuit that mediates the effects of low temperature on fly growth.

## Abstract (web summary)

Animal body size is influenced by climate, but the cellular mechanisms regulating this response are unclear. Here, the authors identify a neuronal circuit composed of a group of cold-sensing neurons and insulin-producing cells that mediates the effects of temperature on *Drosophila* growth.

## Graphical abstract

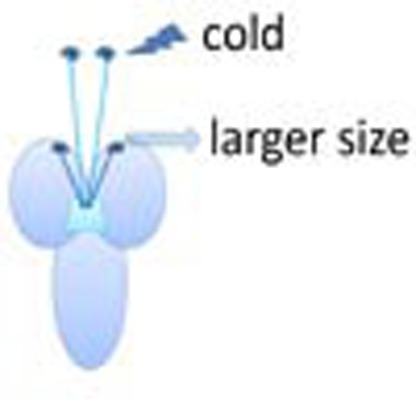

The body size of an animal is influenced by its ecological environment and evolutionary history. Animals in cold climates tend to have bigger body sizes than those in hot climates^1^,^2^,^3^. Temperature has a major impact on *Drosophila* growth and body size^4^,^5^,^6^. Though various explanations for this have been proposed, the cellular and molecular mechanisms for temperature-dependent regulation of body size remain unclear.

Insulin-producing cells (IPCs), functionally analogous to mammalian pancreatic islet β cells, are medially located in the central brain. IPCs play a key role in regulating *Drosophila* growth and metabolism. Blocking or ablation of IPCs results in severely reduced body size and delayed development^7^,^8^,^9^. IPCs sense nutrient signal via fat bodies, leading to release of factors regulating growth and development^9^,^10^. In *Drosophila melanogaster*, there are eight *Drosophila* insulin-like peptides (*dilps*) that regulate growth, metabolism, longevity, fecundity and maturation^11^,^12^. Four of these, *dilp1*, *dilp2*, *dilp3 and dilp5*, are primarily synthesized in IPCs^7^,^8^,^13^. Of the eight *dilps*, *dilp2* is the most potent in promoting growth and *dilp6* promotes growth during the non-feeding stage^14^,^15^. The most recently discovered member of the *dilps* family, *dilp8*, represses ecdysone production and postpones pupariation^16^,^17^. There is only one known receptor for *dilps*, the insulin receptor (InR). In nutrient dependent growth, *Drosophila* IIS (insulin/IGF signalling) functions as a linker between nutrients and systemic growth^10^,^12^.

*Drosophila* can sense hot and cold temperatures^18^,^19^. In an adult fly, there are three neurons in each antenna that express transient receptor potential (TRP) family members encoded by *brivido* genes for cold sensing. There are also three heat sensors located in each antenna^19^. Other sensors expressing TRPA1, a TRP family member responsible for sensing and avoiding heat, are located in the brain^20^,^21^. These cold- and heat-sensing neurons project to adjacent protocerebral regions to form a thermotropic map. Downstream thermal projection neurons project to the mushroom bodies, lateral horns and posterior lateral protocerebrum for further sensory processing^22^,^23^. A subgroup of dopaminergic neurons innervating the mushroom bodies are also responsive to temperature shifts^24^. It is known that, for larval cold sensing, some sensory neurons in terminal organs respond to reduced temperatures^25^. In a recent report, three pairs of neurons in larval dorsal organ ganglions (DOGs) were shown to respond to cold temperature and to be required for cold temperature avoidance^26^. Downstream targets of these primary cold-sensing neurons have not been identified. The molecular basis of larval cold sensing also remains to be characterized, though some clues implicate TRP family members^21^,^26^,^27^.

In this study, we find that IPCs are required for cold-induced regulation of *Drosophila* body size. Stimulating a group of larval cold-sensing neurons that directly innervate IPCs is sufficient to activate IPCs, promote synthesis and secretion of *dilp*s and increase body size, thus mimicking effects of cold temperature. Our results identify a mechanism, at the level of neuronal circuitry, for cold regulation of body size in flies.

## Results

### Cold increased fly body size while reducing food intake

**Figure 1:**
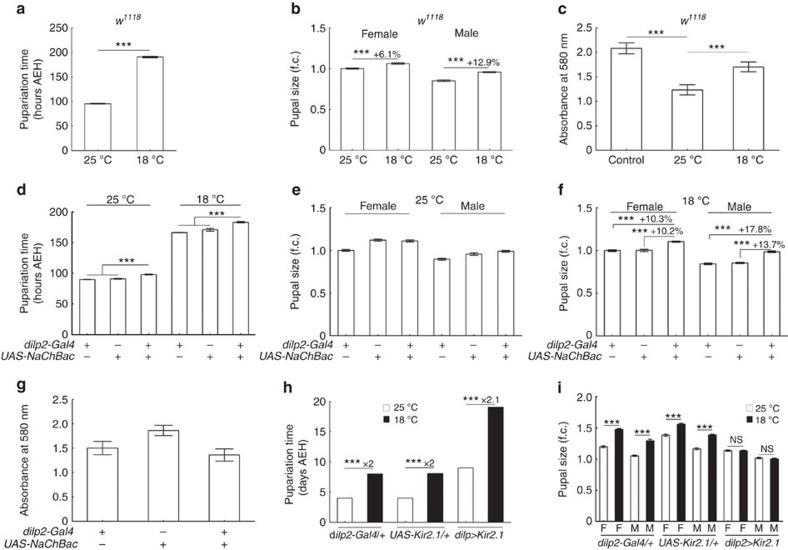
IPCs are required for the effect of low temperature on pupal size. (**a**) Pupariation time of *w*^*1118*^ at 18 °C was later than at 25 °C (*n*=5). (**b**) *w*^*1118*^ pupal size was greater at 18 °C than at 25 °C (*n*=28 for females; *n*=22 for males). (**c**) Absorbance of iodo–starch reactions at 580 nm using residue food from *w*^*1118*^ fly cultures at 25 or 18 °C. Food starch remaining after 25 °C culture was less than after 18 °C culture (*n*=3). See Methods section for further details. (**d**) Pupariation time of larvae expressing NaChBac with *dilp2-Gal4* was later than that of controls both at 25 °C (*n*=7) and at 18 °C (*n*=8). (**e**,**f**) Pupal size of flies expressing NaChBac with *dilp2-Gal4* was larger than that of controls at 18 °C (*n*=24 for both females and males) as shown in **e**, but not at 25 °C (*n*=24 for females; *n*=33 for males) as shown in **f**. (**g**) Absorbance of iodo–starch reaction at 580 nm using residue food after culturing flies expressing NaChBac with *dilp2-Gal4*. Food starch remaining for flies with IPCs hyperactivated was not different from that of controls (*n*=3). (**h**) Pupariation time of larvae expressing Kir2.1 by *dilp2-Gal4* and control similarly increased at 18 °C as compared with at 25 °C (*n*=9). (**i**) Pupal sizes of flies with blocked IPCs cultured at 18 °C were not different from those cultured at 25 °C (*P*>0.05, *n*=13 for both females and males); pupal sizes of controls were significantly larger at 18 °C than at 25 °C (*n*=13). F, females; M, males; AEH, after egg hatching; error bars are s.e.m.; ***P<0.001, Student's *t*-test.

To address the effect of cold temperatures on animal body size, we first measured pupal sizes of flies cultured at 25 and 18 °C. Compared with those raised at 25 °C, *w*^*1118*^ flies raised at 18 °C had significantly larger pupae, a difference found in both genders (Fig. 1b). In addition, pupariation was significantly delayed in *w*^*1118*^ flies raised at 18 °C as compared with those raised at 25 °C (Fig. 1a). This raised the possibility that the larger pupal size at 18 °C was caused by increased food intake during the prolonged larval stage. To address this, we estimated the total food intake of *w*^*1118*^ larvae during the whole larval stage by quantifying food starch, before and after consumption by flies, using an assay based on an iodo–starch reaction (Supplementary Fig. 1). We found that at 25 °C *w*^*1118*^ larvae consumed much more starch and thus more food than at 18 °C, even though the larval stage at 25 °C was much shorter (Fig. 1c). Larval feeding rate, measured during a 20 min period, was also lower in flies raised at 18 °C compared with those at 25 °C (Supplementary Fig. 2). Thus, the increase in pupal size at the cold temperature did not result from increased food intake.

### Cold caused increased fly body size through IPCs

We next investigated how lower temperature promotes increased pupal size. Because IPCs are well known regulators of body size in *Drosophila*^8^,^9^, we examined whether temperature-dependent body size effects involve IPCs. We expressed the depolarizing Na^+^ channel NaChBac (ref. 28) in IPCs with *dilp2-Gal4* to determine whether activation of IPCs would have effects on pupal size and pupariation comparable to those of low temperature. We found that increasing excitability of IPCs in flies delayed pupariation at both 18 and 25 °C, but pupal size was increased only at 18 °C (Fig. 1d–f). On the basis of total food intake measurements, flies expressing *UAS-NaChBac* in IPCs did not consume more food than control flies when both were reared at 18 °C (Fig. 1g). We used optogenetic tools to verify the relationship between activation of IPCs and *Drosophila* growth. Directly activating IPCs, with exposure to 620 nm red light, in flies expressing *UAS-Chrimson* (ref. 29) with *dilp2-Gal4* resulted in significantly increased pupal size (Supplementary Fig. 3). We then tried to block IPCs using *UAS-Kir2.1*, a potassium channel that can hyperpolarize neurons^30^, to determine whether it abolished cold regulation of pupal size. Unexpectedly, blocking IPCs with *UAS-Kir2.1* in flies did not lead to a change in pupal size relative to that of control flies when both were cultured at 25 °C. However, when flies were cultured at 18 °C, those expressing *UAS-Kir2.1* had significantly smaller pupal sizes than the controls (Supplementary Fig. 4). Further examination of the data revealed that the pupal sizes of flies with IPCs blocked by Kir2.1 were unaffected by temperature shift, whereas in control flies pupal sizes were significantly larger when reared at 18 °C versus at 25 °C (Fig. 1i). The pupal size increase in these transgenic control flies appeared to be more significant than in *w*^*1118*^, which might reflect the involvement of genetic factors in cold regulation of pupal size. Interestingly, like in controls, the pupariation time of IPCs-blocked flies at 18 °C was roughly twice that at 25 °C (Fig. 1h) suggesting that pupariation time was not affected by blocking IPCs. These results suggest that cold-dependent regulation of *Drosophila* body size, but not of pupariation time, depends on IPCs.

### Cold-activated IPCs and affected *dilps*

**Figure 2:**
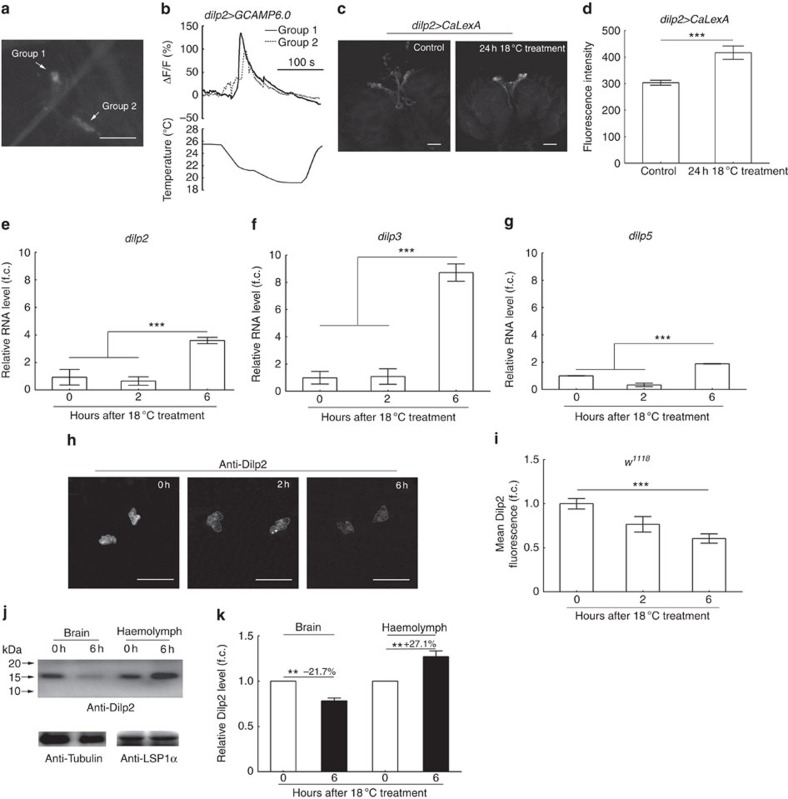
IPCs are responsive to cold temperature. (**a**,**b**) Ca^2+^ imaging of IPCs responses to a temperature decrease. The responses of two groups of IPCs on each side of the brain are shown as representatives. (**c**) CaLexA-based imaging of larval IPCs after 24 h culture at 18 °C. (**d**) Quantification of **c** (*n*=7). (**e**–**g**) Culturing *w*^*1118*^ larvae at 18 °C for 6 h increased mRNA expression levels of *dilp2*, *dilp3* and *dilp5*. Fold changes are relative to conditions at 0 h (*n*=3). (**h**) Anti-Dilp2 staining in IPCs of *w*^*1118*^ larvae raised on poor food was lower after 18 °C treatment. (**i**) Quantification of **h** (*n*=20). (**j**,**k**) Dilp2 levels decreased in brain but increased in haemolymph after 6 h of 18 °C treatment. (**j**) Western blotting of brain and haemolymph Dilp2. (**k**) Quantification of **j** (*n*=3). Scale bars, 50 μm for all; error bars are s.e.m.; **P<0.01, ***P<0.001, Student's *t*-test or analysis of variance.

To seek direct confirmation of the putative relationship between cold stimulation and IPCs, we first examined whether IPCs respond to cold using calcium (Ca^2+^) imaging. Ca^2+^ sensitive GCAMP6.0 (ref. 31) was expressed in IPCs to monitor cellular activity in response to a temperature decrease. Decreasing the temperature from 25.5 to 18 °C produced a strong response in all IPCs (Fig. 2a,b and Supplementary Movie 1). In contrast, IPCs did not respond to a temperature increase from 25 to 30.5 °C (Supplementary Fig. 5 and Supplementary Movie 2). In addition, we used an NFAT-based neural tracing method, CaLexA (calcium-dependent nuclear import of LexA)^32^, to measure response of IPCs to long-term cold treatment. A 24-h exposure to 18 °C resulted in significantly higher level of activity-dependent green fluorescent protein (GFP) accumulation in IPCs than in cells at 25 °C (Fig. 2c,d). Together, these findings showed that IPCs respond to both acute and chronic exposure to cold.

We next examined whether more specific molecular events in IPCs are affected by cold stimulation. In previous studies, nutrient-induced effects on IPCs were measured by transcription levels of *dilps* genes and secretion of Dilps protein^7^,^9^. In these reports, starvation suppressed *dilp3* and *dilp5* transcription and Dilp2 secretion in IPCs. We employed similar methods to measure effects of cold temperature on IPCs. We exposed 25 °C-reared *w*^*1118*^ larvae to 18 °C for various periods of time (0, 2 and 6 h). Quantitative real-time PCR showed that, at 6 h, expression levels of *dilp2*, *dilp3* and *dilp5* in larval central nervous system were increased with *dilp3* most significantly (Fig. 2e–g). The elevated expression of these genes was no longer evident after cold exposures of 14 or 24 h (Supplementary Fig. 6). We also investigated Dilp2 protein secretion by larval IPCs. Cold exposure for 6 h led to greatly reduced Dilp2 accumulation in *w*^*1118*^ larvae raised on poor food at 25 °C (Fig. 2h,i). Since *dilp2* transcription had been enhanced by low temperature, we postulated that the reduced Dilp2 accumulation reflected increased Dilp2 secretion by IPCs. Indeed, after 6 h of 18 °C treatment Dilp2 levels in haemolymph was increased by 27.1% while brain Dilp2 levels were decreased by 21.7% (Fig. 2j,k and Supplementary Fig. 7). Thus, low temperature exposure promotes *dilp2*, *dilp3 and dilp5* transcription and Dilp2 secretion in fly larvae. We also examined transcription of other *dilps*. Quantitative real-time PCR showed no significant changes in global mRNA expression of a number of *dilps (dilp1*, *dilp4*, *dilp6 and dilp7)* after various periods of 18 °C treatment. However, a twofold increase was seen in *dilp8* (Supplementary Fig. 8). Although *dilp8* has been reported to postpone pupariation by repressing ecdysone synthesis, its overexpression is unable to enhance body size^16^,^17^. Fat bodies are the major sites for synthesis of *dilp6*, which is capable of promoting nutrient-independent fly growth during the non-feeding stage^14^,^15^. However, we did not detect a significant response of fat body cells to cold treatment by Ca^2+^ imaging (Supplementary Fig. 9).

Taken together, these data showed that low temperature can activate IPCs, promoting transcription of *dilp2*, *dilp3*, *dilp5* genes as well as secretion of Dilp2 protein.

### Activation of cold-sensing neurons enhanced fly body size

**Figure 3:**
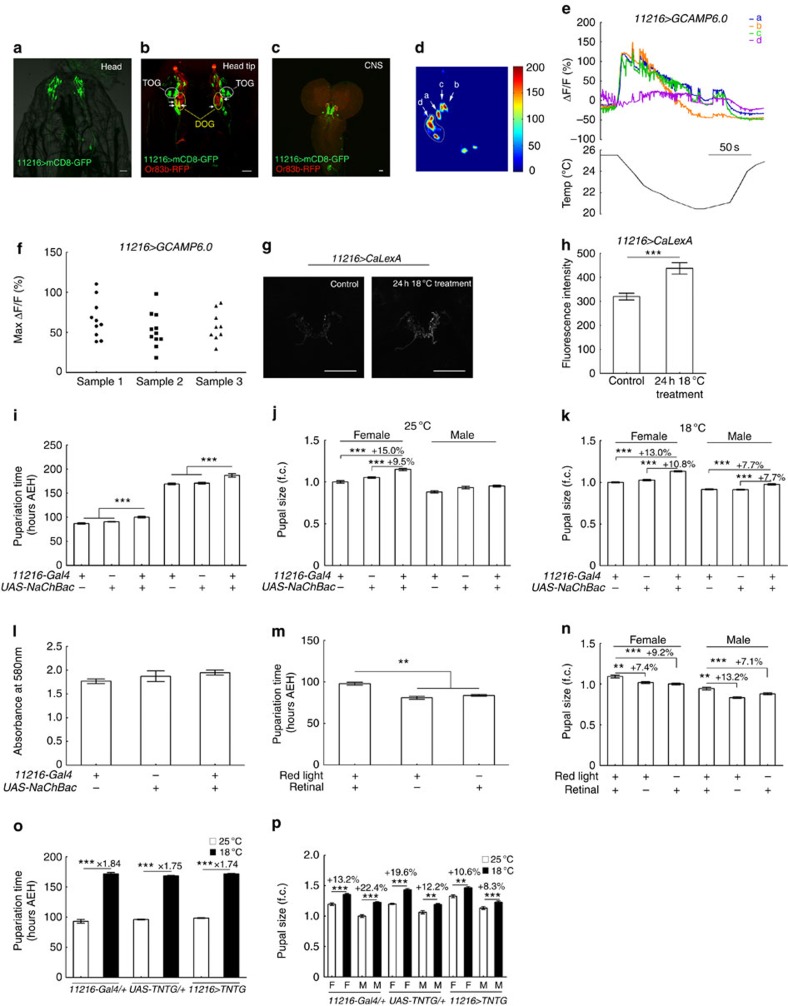
*11216-Gal4* labels cold-sensing neurons in larval flies. (**a**–**c**) Expression of *11216-Gal4* and *Or83b-RFP* in larval head and central nervous system. Arrows indicates the DOG neurons. Note that the two rightmost DOG neurons are overlaid in **b**. Scale bars, 20 μm. (**d**,**e**) Ca^2+^ imaging of *11216-Gal4* neurons in response to a temperature decrease. Imaging of *11216-Gal4* neurons in one representative sample is shown as a heat map (**d**) and corresponding fluorescence intensity curves (**e**). Four different groups of neurons are designated a, b, c and d. (**f**) The scatter plot shows peak responses of *11216-Gal4* neurons to ice water in three representative samples. (**g**) CaLexA-based imaging of the axonal termini of *11216-Gal4* neurons after 24 h culture at 18 °C. Scale bars, 50 μm. (**h**) Quantification of **g** (*n*=7). (**i**) Activation of *11216-Gal4* neurons by overexpressing NaChBac-delayed pupariation at both 25 °C (*n*=6) and 18 °C (*n*=6). (**j**) At 25 °C, pupal sizes of flies with *11216-Gal4* neurons activated by overexpressing NaChBac were larger than in parental controls for females but not for males (*n*=29 for females; *n*=21 for males). (**k**) At 18 °C, an increase in pupal size was seen in both females and males (*n*=21 for females; *n*=19 for males). Fold changes are relative to the average pupal size of female *11216-Gal4* flies. (**l**) Absorbance of iodo–starch reaction at 580 nm using residue food after culturing flies expressing *UAS-NaChBac* by *11216-Gal4*. Food starch after culturing *11216-Gal4* neurons-activated flies was not different from that of controls (*n*=3). (**m**,**n**) Optogenetically activating *11216-Gal4* neurons delayed pupariation (**m**) and enhanced pupal size in both females and males (**n**; for pupariation, *n*=4; for pupal size, *n*=21 for females, *n*=18 for males.) (**o**,**p**) Pupariation time (**o**) and pupal sizes (**p**) of larvae with ectopic expression of TNTG by *11216-Gal4* and of controls increased similarly at 18 °C as compared with at 25 °C (for pupariation, *n*=8; for pupal size, *n*=17 for both females and males). F, females; M, males; AEH, after egg hatching; error bars are s.e.m.; **P<0.01, ***P<0.001, Student's *t*-test.

In speculating on how low temperature signals might reach IPCs and affect their activity, the most likely transmitters are larval cold-sensing neurons. By screening ∼1,000 Gal4 lines, we obtained one line numbered 11216, in which neurons morphologically similar to the previously reported cold-sensing neurons were labelled^25^,^33^,^34^ (Fig. 3a). There were about 9 to 10 pairs of neurons (named *11216-Gal4* neurons) labelled in the anterior tip of larval head. To distinguish subsets of *11216-Gal4* neurons, we compared the expression pattern of *11216-Gal4* with that of Or83b-RFP, which labels all larval odour receptor neurons and thus marks the positions of DOGs where the cell bodies of odour receptor neurons are (Fig. 3b,c). *11216-Gal4*-labelled three pairs of neurons in the DOGs but with no overlap with Or83b-RFP signal. Dendrites of these DOG neurons seemed to innervate somewhere medial and posterior to the terminal organs, but not the terminal organs themselves. Outside of the DOGs, *11216-Gal4* also labelled about five pairs of terminal organ ganglion (TOG) neurons, which sent out dendrites to innervate terminal organs. There were other one to two pairs of neurons with cell bodies located posterior to the DOGs. Their dendritic termini intermingled with other neurons and were hard to identify. In addition to the cells in head tip, about four pairs of neurons in the pharyngeal region and intestinal cells were labelled in this Gal4 line (Fig. 3a, Supplementary Fig. 10 and Supplementary Table 1). We used Ca^2+^ imaging to confirm that this Gal4 labels cold-sensing neurons. By expressing GCAMP6.0, we found that at least 30% of the imaged *11216-Gal4* neurons responded to a temperature decrease from 25 to 20 °C with the strongest response to be a 100% increase in fluorescence intensity (Fig. 3d,e and Supplementary Movie 3). The *11216-Gal4*-labelled DOG neurons appeared to be more sensitive to temperature decrease than the TOG neurons. These neurons even responded robustly to a small temperature decrease from 25 to 24 °C (Supplementary Fig. 11). When subjected to an abrupt temperature drop from 25 to14 °C, almost all the imaged *11216-Gal4* neurons showed at least some activation (Fig. 3f). Notably, none of the *11216-Gal4* neurons imaged in the larval anterior terminal region responded to a temperature increase from 25 to 30 °C (Supplementary Fig. 12 and Supplementary Movie 4). In addition, there was no detectable response of *11216-Gal4*-labelled intestinal and pharyngeal cells to cold by Ca^2+^ imaging (Supplementary Figs 13 and 14). The responsiveness of *11216-Gal4* neurons was further confirmed with the NFAT-based CaLexA technique. A 24-h exposure to 18 °C, as compared with 25 °C, resulted in significantly higher levels of activity-dependent GFP accumulation in the axonal termini of *11216-Gal4*-labelled neurons (Fig. 3g,h). Thus, *11216-Gal4* indeed labels cold-sensing neurons.

We then examined whether activating these cold-sensitive *11216-Gal4* neurons could mimic the effect of low temperature on pupal size and pupariation time. We hyperactivated these neurons by overexpressing NaChBac (using *UAS-NaChBac*) with *11216-Gal4* and found that pupariation was significantly delayed in flies with *112162-Gal4* neurons activated as compared with control flies, at both 18 and 25 °C (Fig. 3i). Flies of both genders with *11216-Gal4* neurons activated had significantly larger pupal sizes than controls at 18 °C (Fig. 3k). We estimated total food intake during the whole larval stage using the iodo–starch assay. Flies reared at 18 °C with *UAS-NaChBac* expressed in *11216-Gal4* neurons did not consume more food than the controls reared at the same temperature (Fig. 3l). At 25 °C, increased pupal size was evident in females, but not in males (Fig. 3j). We also activated the *11216-Gal4* neurons optogenetically by expressing *UAS-Chrimson* and exposing them to 620 nm red light. Optogenetic activation led to significantly delayed pupariation (Fig. 3m) and increased pupal sizes (Fig. 3n). On the other hand, we tried to inhibit *11216-Gal4* neurons with TNTG, a tetanus toxin that prevents neurotransmission in neurons^35^. The cold-induced delay in pupariation time of flies with *11216-Gal4* neurons inhibited by TNTG was roughly the same as that of controls (Fig. 3o). Pupal size after a temperature decrease from 25 to 18 °C was also significantly increased, though to a lesser extent than in the control flies (Fig. 3p). Pupal sizes of 18 °C-reared flies with *11216-Gal4* neurons expressing the inhibitory *UAS-TNTG* were not different from the controls (Supplementary Fig. 15). These results suggest that blocking *11216-Gal4* neurons did not substantially undermine cold regulation of body size. In addition to *11216-Gal4*, we also tested other Gal4 lines, *GH86-Gal4* (ref. 25) and *iav-Gal4* (ref. 27), that label neurons involved in cold-related *Drosophila* behaviours to see if the these neurons contribute to the effects of cold on growth. When cultured at either 18 or 25 °C, flies with these neurons hyperactivated with *UAS-NaChBac* did not show larger pupal size than corresponding non-hyperactivated controls (Supplementary Figs 16 and 17). This suggests that these neurons are not involved in the cold regulation of *Drosophila* growth. Together, these findings support the involvement of *11216-Gal4*-labelled cold-sensing neurons in regulation of *Drosophila* body size by low temperature.

### Cold-sensing neurons activated IPCs and affected *dilps*

**Figure 4:**
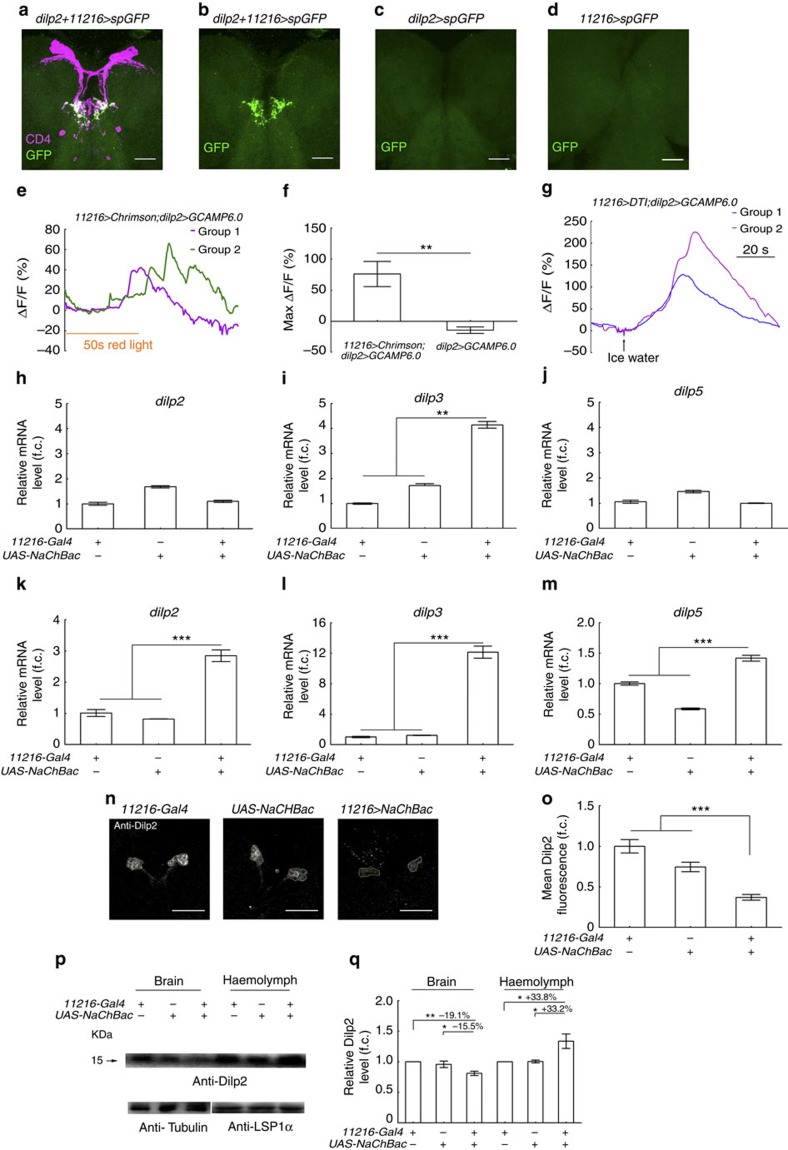
Larval *11216-Gal4* neurons directly interact with IPCs. (**a**–**d**) A strong GRASP signal was seen between *11216-Gal4* neurons and IPCs but no detectable signal was seen in controls. Magenta, anti-CD4 (labels both *11216-Gal4* neurons and IPCs); green, GFP (GRASP signal). Scale bars, 10 μm. (**e**) Ca^2+^imaging of IPCs when *11216-Gal4* neurons expressing Chrimson were activated by 620 nm red light. The IPCs on both sides of the brain showed asynchronous responses. Groups 1 and 2 are two groups of IPCs on each side of the brain. (**f**) The histogram shows the average maximum increase in fluorescence intensity in experimental and control samples (*n*=7). (**g**) Ca^2+^ imaging of the response of larval IPCs to ice water in flies with *11216-Gal4* neurons ablated with Diphtheria toxin (DTI). In a representative sample, two groups of IPCs on each side of the brain showed strong responses. (**h**–**m**) Larvae with *11216-Gal4* neurons activated by NaChBac showed higher *dilp2* and *dilp5* mRNA levels at 18 °C but not 25 °C, while *dilp3* mRNA was always higher than controls at both 18 and 25 °C. Fold changes are relative to mRNA levels in *11216-Gal4* larvae (*n*=3). (**n**–**o**) Anti-DILP2 staining signal in poor food-raised flies with *11216-Gal4* neurons activated by NaChBac was lower than in controls. Fold changes are relative to level in *11216-Gal4* (*n*=17). Scale bars, 50 μm. (**p**,**q**) Dilp2 levels decreased in brain but increased in haemolymph when *11216-Gal4* neurons were activated by NaChBac. (**p**) Western blotting of brain and haemolymph Dilp2. (**q**) Quantification of **p** (*n*=3). Error bars are s.e.m.; *P<0.05, **P<0.01, ***P<0.001, Student's *t*-test.

We next examined whether these *11216-Gal4*-labelled cold-sensing neurons directly target IPCs. As shown in Fig. 3c the axons of *11216-Gal4* neurons terminated in the subesophageal ganglion (SOG) region of the brain. Interestingly, IPCs also have dendritic arborizations in the SOG region^8^. Therefore, we tested possible interactions between these two groups of neurons using the GFP reconstitution across synaptic partners (GRASP) technique^36^,^37^,^38^. A significant GRASP signal was observed between IPCs labelled with *dilp2-LexA* and *11216-Gal4* neurons while no such signal was seen in controls that expressed only one of the two split GFPs (Fig. 4a–d). This suggested that the axonal termini of *11216-Gal4* neurons directly interact with IPCs in the SOG region. To confirm the existence of a functional connection, we combined optogenetic and Ca^2+^ imaging techniques to determine whether directly exciting *11216-Gal4* neurons activates IPCs^39^. The *11216-Gal4* neurons were excited by *UAS-Chrimson* on red-light stimulation^29^. Simultaneously, Ca^2+^ imaging using *LexAop-GCAMP6.0* (ref. 31) under control of *dilp2-LexA* revealed a strong Ca^2+^ response in IPCs on a 50-sec stimulation with red light (Fig. 4e,f and Supplementary Movie 5). The same red-light stimulation applied to control larvae that did not carry *UAS-Chrimson* and *11216-Gal4* was unable to induce a Ca^2+^ response in IPCs (Supplementary Fig. 18 and Supplementary Movie 6). Nonetheless, ablating the *11216-Gal4* neurons with *UAS-DTI* (ref. 40) did not inhibit the response of IPCs to cold stimulation (Fig. 4g) suggesting that *11216-Gal4* neurons are not the only pathway linking cold temperature to IPCs. Taken together, these results showed that the *11216-Gal4*-labelled cold-sensing neurons directly activate IPCs.

We further set out to ask whether activation of the *11216-Gal4*-labelled cold-sensing neurons promoted the transcription of *dilps* and secretion of Dilp2 as cold temperature exposure did. Quantitative PCR was used to measure relative expression levels of *dilp 2*, *dilp3*, and *dilp5* in larvae overexpressing NaChBac with *11216-Gal4*. At 25 °C, *dilp3* mRNA levels were significantly higher in larvae overexpressing NaChBac with *11216-Gal4* than in controls, whereas such difference was not seen in *dilp2* and *dilp5* mRNA levels (Fig. 4h–j). However, at 18 °C, mRNA levels of *dilp2*, *dilp3* and *dilp5* were all significantly elevated in larvae with *11216-Gal4* neurons activated as compared with in controls (Fig. 4k–m). Optogenetic activation of the *11216-Gal4* neurons also resulted in elevated expression of *dilp2*, *dilp3 and dilp5* (Supplementary Fig. 19). Interestingly global *dilp8* level was also significantly increased on optogenetic activation of *11216-Gal4* neurons while level of *dilp6* was not (Supplementary Fig. 20). This is consistent with our observation that cold treatment enhanced expression of *dilp8* but not *dilp6*. We next examined Dilp2 secretion from IPCs. Poor food-cultured larvae with *11216-Gal4* neurons hyperactivated by NaChBac showed a significantly lower level of Dilp2 in IPCs than in the controls (Fig. 4n,o). Consistent with this, level of Dilp2 in larval haemolymph was significantly higher in these flies than in the controls. Haemolymph Dilp2 level was increased by >33% while brain Dilp2 level decreased by >15% (Fig. 4p,q). These results show that activation of the *11216-Gal4*-labelled cold-sensing neurons can promote expression of *dilps* and secretion of Dilp2 in IPCs, similar to the effects of cold temperature exposure.

## Discussion

Our findings show that flies attain larger body sizes at lower temperatures without increased food consumption. Cold temperature is detected by a group of *11216-Gal4*-labelled cold-sensing neurons that, in turn, activate IPCs to synthesize growth-promoting hormones such as *dilp2*, *dilp3*, *and dilp5*. Thus, we have identified a neuronal circuit-based mechanism of body size regulation by cold temperature.

One potential concern about cold regulation of body size is that it may be a secondary effect of nutrient-regulated growth. We eliminated this possibility with two findings. First, as previously reported^41^, *Drosophila* larvae eat less at lower temperatures when food consumption is measured during short time periods. We used a simple 20-min dye ingestion assay to confirm this (Supplementary Fig. 2). This eliminated the possibility that acute increases in nutrient signalling enhanced growth and body size at cold temperatures. Second, we used an assay based on iodo–starch reaction to estimate total food intake during the entire larval stage. We found that total food intake was reduced in larvae raised at 18 °C, compared with those raised at 25 °C. This excluded the possibility that cumulative nutrient signalling stimulates growth at lower temperatures. Therefore, cold-induced *Drosophila* growth is not caused by increased consumption of nutrients. In our study, cold regulation of pupal size seemed to be independent of *dilp6*, a known regulator of fly growth during the non-feeding stage^14^,^15^, since cold treatment caused no change in *dilp6* expression. It is, nonetheless, possible that cold regulation of body size shares some of the same downstream targets as *dilp6* in regulating nutrient-independent growth.

IPCs are known to be responsive to nutrient signals^9^,^10^. We found that IPCs are also required for the cold enhancement of pupal size occurring without increased food intake. The cellular and molecular events in IPCs induced by cold and nutrient signalling are quite similar, including upregulation of *dilp3* and *dilp5* expression and enhanced Dilp2 secretion. One difference is that *dilp2* transcription, though upregulated by cold stimulation, is not affected by nutrient signals. Thus, it is likely that cold-induced and nutrient-induced growth share common cellular and molecular mechanisms.

Notably, the delayed larval pupariation we observed at the colder temperature appeared to be largely independent of IPCs. Though activating IPCs led to slightly delayed pupariation, blocking IPCs with Kir2.1 had little effect on the cold-induced delay in pupariation, while completely abolishing the increase in pupal size. This indicates that distinct mechanisms regulate the delay in pupariation and the enhancement in pupal size induced by cold temperature. Since *dilp8* inhibits ecdysone synthesis and larval development^16^,^17^ and, in our hands, showed increased expression after both cold treatment and activation of the cold-sensing *11216-Gal4* neurons (Supplementary Fig. 19), it might serve as the molecular mediator of cold-induced developmental delay. However, more work will be needed to clarify this issue.

In this study, we identified *11216-Gal4* neurons that can sense cold and, in turn, activate IPCs. These neurons are different from the known larval cold-sensing neurons labelled by *GH86-Gal4* (ref. 25) since activating *GH86-Gal4*-labelled neurons was not sufficient to increase pupal size. Other neurons, such as *iav*-expressing neurons reported to be involved in cold-related effects^27^, also lack growth-promoting capacity. Taken together, these findings indicate that *11216-Gal4*-labelled neurons transmit specific ‘growth-promoting' cold signals. It is worth noting that *11216-Gal4*-labelled neurons are not homogenous: one subgroup of neurons, likely DOG neurons, is sensitive to subtle temperature drop, while other subsets of neurons, mostly TOG neurons, respond only to larger decrease in temperature. Although we are unable to tell which subgroup of neurons is involved in cold regulation of growth and which is not, it is conceivable that their contributions to growth regulation are different. On the other hand, we found that blocking *11216-Gal4* neurons did not prevent the cold-induced increase in pupal size. Thus, other, as yet unidentified, cold-sensing neurons must also play a significant role in transmitting cold signals to IPCs and, thereby, modulating growth.

The significance to the animal of cold-induced stimulation of IPCs and expression of *dilps* is an intriguing question. Typically, metabolism is slower in animals at lower temperatures, though insulin generally functions as a signal to promote metabolism. One explanation for this apparent paradox is that at cold temperatures the metabolic rate, including in ectotherms like *Drosophila*, is decreased such that energy supply is insufficient for normal demand. The cold-induced stimulation of IPCs then serves as a forced way to increase insulin levels and, thereby, metabolic rate and energy stores, enabling basal level energy demands to be met. Concomitantly, animal growth is also stimulated. In this model, the response of IPCs to cold can thus be regarded as a compensation for reduced metabolism, improving survival of the animal at cold temperatures.

In summary, our discovery not only provides an explanation, based on neural circuitry, for the regulation of animal body size by low temperatures but also provides new insights into how the environment affects animal physiology.

## Methods

### Fly strains and food

Flies were reared on a standard cornmeal medium at 25 °C under a 12 h:12 h light:dark cycle. The restricted diet (poor food) for assaying Dilp2 secretion corresponded to 0.1 × medium^9^. The following strains were used: *w*^*1118*^; *11216-Gal4*; *dilp2-KO*^11^; *dilp2-Gal4* (ref. 8); *dilp2-LexA*; *cg-Gal4* (ref. 42); *GH86-Gal4* (ref. 25); *iav-Gal4* (ref. 27); *Or83b-RFP*□*LexAop-GCAMP6.0* (ref. 31); *LexAop-mCD4-spGFP11* (refs 37, 38); *UAS-mCD4::spGFP1-10* (refs 37, 38); *UAS-mCD8-GFP* (ref. 43); *UAS-GCAMP6.0* (ref. 31); *UAS-Chrimson* (ref. 29); *UAS-NaChBac* (ref. 28); *UAS-Kir2.1* (ref. 30); *UAS-TNTG* (ref. 35); *UAS-DTI* (ref. 40) and *LexAop-CD2-GFP*; *UAS-mLexA-VP16-NFAT*, *LexAop-CD8-GFP-2A-CD8-GFP* (ref. 32).

### Generation of transgenic flies

An 865-bp fragment of the 5′-regulatory region of *dilp2* and the LexA::VP16::SV40 sequence were each amplified from the genomic DNA of *UAS-mCD8::GFP* and *Or83b-LexA::VP16*. The following PCR primers with specific restriction sites were used to amplify the corresponding sequences:

dilp2F: 5′-CCAACACACACACATTC-3′;

dilp2R: 5′-TGGTTATGGGTTTACTG-3′;

LexA::VP16F: 5′-CCAAGCTTATGAAAGCGTTAACGGCCAG-3′;

LexA::VP16R: 5′-CGCCTAGGCTACCCACCGTACTCGTCAA-3′;

SV40F: 5′-AGGCGGCCGCGATCTTTGTGAAGGAACCTTACTTC-3′;

SV40R: 5′-AGCTGCAGGATCCAGACATGATAAGATACATTGA-3′.

The PCR products were first cloned into a PMD-18 T vector (Takara Bio. Inc. Otsu, Shiga, Japan). The KpnI/NotI fragment containing the *dilp2* promoter region^8^ was sequentially joined with the NotI/SpeI fragment containing LexA::VP16 and the SpeI/XbaI fragment containing SV40 before final insertion into the KpnI/XbaI site of pCaSpeR4. The recombinant plasmid was then germline transformed to obtain transgenic *dilp2-LexA* flies.

### Iodo–starch assay for food intake measurement

Food intake was indirectly measured by quantifying food starch before and after *Drosophila* culture with a method based on iodo–starch reaction^44^. Larvae, first instar (for *w*^*1118*^) or second instar (for crosses with *UAS-NaChBac*, which was balanced with *TM6b*,*Tb*), were transferred to vials (20 larvae per vial) each containing 1 ml of food. For controls no larvae were placed in the vial. In all samples, the female/male ratio was about 1:1, excluding potential effects of sexual dimorphism on food intake. After pupariation, all pupae were removed from each vial. The food in each vial was air dried in a 37 °C incubator for 24 h, then removed and weighed. All food from each vial was then added to 70 ml dH_2_O and boiled for 20 min. The food solution was allowed to cool to room temperature and adjusted to a final volume of 50 ml with dH_2_O. The food solution was then serially diluted in a total volume of 50 μl. These samples were then mixed with 50 μl KI-I_2_ solution (5 mM I_2_ and 5 mM KI) and absorbance read from 500 to 700 nm, at 20 nm intervals, using a Varioskan Flash spectral scanning multimode reader (Thermo Fisher Scientific Inc. Waltham, MA USA). Absorbance values were linear in the range of the serial dilutions (Supplementary Fig. 1). Absorbance values of 1:1 dilution at 580 nm, the maximum absorbance, were used to indicate food starch concentration.

### Dye-feeding assay

Synchronized 72–74-h AEL larvae were carefully isolated from food medium and washed with PBS. Groups of 10 larvae were placed in 2 ml yeast paste (0.5 g of yeast powder per millilitre water, 0.05% food dye (FD&C Blue No.1, Sigma-Aldrich, St. Louis, MO, USA)) on 1.5% agar plate preincubated at the indicated temperatures. Larvae were allowed to feed for 20 min before being washed and photographed.

### Fly culture for optogenetics

Larvae used for optogenetic experiments were raised at 25 °C in constant darkness on food supplemented with 200 nM all-trans-retinal. In experiments involving optogenetic activation of neurons during development, light-emitting-diode-emitted red light (620±15 nm) was applied to the flies throughout the culturing period to stimulate the relevant neurons. For activation of IPCs, light pulses were for periods of 60 s ON:120 s OFF; for activation of *11216-Gal4* neurons, light pulses were for periods of 20 s ON:20 s OFF.

### Measurement of pupal size and pupariation time

Eggs were collected on apple-juice agar plates for 2 h. In each experiment, 40 newly hatched first-instar larvae were collected 24 h after egg laying and transferred to vials, each containing ∼5 ml of standard cornmeal food or food supplied with 200 nM all-trans-retinal for optogenetic experiments. Pupariation time was scored every 3–8 h as the time that new pupae appeared. The pupae were then washed and photographed. The images were processed with ImageJ (http://imagej.nih.gov/ij/) to measure the relative areas of pupae as an indicator of pupal size. Larval gender was distinguished by placing each individual in a single well of a 96-well plate and determining the sex of the enclosed adult. Pupariation time was measured *en masse* for females and males since gender influence on pupariation is minimal^45^. The pupal sizes of female and male flies were quantified separately. It should be noted that at the two culture densities employed, 40 larvae per 5 ml food vial (for normal conditions) and 20 larvae per 1 ml food vial (for food intake measurements), pupal size and pupariation time appeared to be the same at least for *w*^*1118*^ (Supplementary Fig. 21).

### Cold treatment of larvae

Third-instar larvae raised at 25 °C were transferred to vials that had been pre-cooled in a 18-°C incubator. After various incubation times (0, 2, 6, 14 or 24 h), larvae (∼76 h after egg hatching) were removed and washed in PBS before being processed for RNA extraction and quantitative reverse transcription (RT)-PCR.

### Western blotting

Brain or haemolymph protein extracts were obtained from 30 larvae. The larvae were collected and dissected in cold PBS then homogenized in lysis buffer (50 mM Tris-HCl, pH 7.5, 150 mM NaCl, 1 mM EGTA, 1% NP-40, and Complete Roche protease inhibitor (Roche Inc., Mannheim, Germany)). Samples were denatured in 5 × SDS loading buffer (containing 1 mM dithiothreitol) and boiled for 5 min. Aliquots of samples were loaded onto a 12% SDS-glycine-polyacrylamide gel. After separation, proteins were transferred to 0.22 μm Immobilon-P membrane (Millipore; Billerica, MA, USA). Membranes were blocked in 5% nonfat dried milk in phosphate-buffered saline with Tween 20 for 30 min and incubated with rabbit anti-Dilp2 (1:3,000) or anti-alpha-Tubulin (1:3,000, ab52866, Abcam Inc., Cambridge, UK) or anti-LSP1α (1:5,000, P11995, Abmart Inc., Berkeley Heights, NJ, USA) at room temperature for 4 h with gentle shaking. Anti-alpha-tubulin was used as control for brain. Anti-LSP1α was used as control for haemolymph (Geminard *et al*., 2009). The membrane was rinsed three times with phosphate-buffered saline with Tween 20 for 10 min and incubated at room temperature for 30 min with horseradish peroxidase (HRP)-conjugated secondary antibody (1:10,000). Enhanced chemiluminescence (Westar Supernova XLS30020, Cyanagten Inc. Bologna, Italy) was used to detect the primary antibodies.

To verify the specificity of anti-Dilp2 in the western blot, whole body protein extractions were prepared from 30 *w*^*1118*^ or *dilp2-KO* adult flies^11^. Sample preparation and analysis procedures were the same as described above for larval samples. The anti-Dilp2 antibody showed good specificity for Dilp2 in western blots (Supplementary Fig. 7).

### Quantitative RT–PCR

Third-instar larvae (∼76 h after egg hatching) were synchronized for 2 h before the brains were dissected out and flash-frozen. Total RNA was extracted using an RNeasy Mini Kit (Qiagen Inc. Venlo, Limberg, Netherlands) according to the manufacturer's instructions. cDNA was reverse transcribed using a Transcriptor First Strand cDNA Synthesis Kit (Roche Inc., Mannheim, Germany). We used FastStart Universal SYBR Green Master /ROX qPCR Master Mix (Thermo Fisher Scientific Inc., Waltham, MA USA) to conduct RT–PCR and *act88* (actin) was used as the control. Data were collected from triplicate measurements. Relative differences in *dilp1-8* gene expression levels were quantified as 2^ΔCt^ (ΔCt is the Ct value of the gene of interest subtracted from the Ct value of actin).

The following primers were used to amplify *dilps* and *actin*:

Dilp1F: 5′-AATGGCAATGGTCACGCCGACTGG-3′;

Dilp1R: 5′-GCTGTTGCCCAGCAAGCTTTCACG-3′;

Dilp2F: 5′-AGCAAGCCTTTGTCCTTCATCTC-3′;

Dilp2R: 5′-ACACCATACTCAGCACCTCGTTG-3′;

Dilp3F: 5′-TGTGTGTATGGCTTCAACGCAATG-3′;

Dilp3R: 5′-CACTCAACAGTCTTTCCAGCAGGG-3′;

Dilp4F: 5′-TGGATTTACACGCCGTGTCAGGCG-3′;

Dilp4R: 5′-ACACCCTTCTCCGTATCCGCATGG-3′;

Dilp5F: 5′-GAGGCACCTTGGGCCTATTC-3′;

Dilp5R: 5′-CATGTGGTGAGATTCGGAGC-3′;

Dilp6F: 5′-TGCTAGTCCTGGCCACCTTGTTCG-3′;

Dilp6R: 5′-GGAAATACATCGCCAAGGGCCACC-3′;

Dilp7F: 5′-GAGCTGTACTCCTGTTCGTCCTGC-3′;

Dilp7R: 5′-TCCAAGCCTCATCATTGCCCGTCC-3′;

Dilp8F: 5′-CGACAGAAGGTCCATCGAGT-3′;

Dilp8R: 5′-GATGCTTGTTGTGCGTTTTG-3′;

Act88F: 5′-AGGGTGTGATGGTGGGTATG-3′;

Act88R: 5′-CTTCTCCATGTCGTCCCAGT-3′.

### Calcium imaging

At room temperature (25 °C), fly larvae were dissected in adult haemolymph-like solution^25^ to remove the posterior half. The remaining part was immobilized under a coverslip. The sample was placed on a glass slide in the holder of a temperature controller (CL-100, Warner Instruments, Hamden, CT, USA), so that temperature changes could be monitored and controlled. Temperature decreases from 25 to 18 °C and temperature increases were achieved using an In-line Heater/Cooler (SC-20, Warner Instruments, Hamden, CT, USA). A rapid temperature drop from 25 to 14 °C was achieved by adding 200 μl of ice water to the head of the larva.

Imaging of neuronal responses was performed on a Nikon Eclipse *Ti*-S fluorescence microscope with a × 20 objective lens. Images were acquired at 2.56 frames per second.

For quantitative analysis of Ca^2+^ imaging data, images were batch processed with ImageJ to determine fluorescence intensity of regions of interest, primarily cell bodies of neurons. Ten sequential images before the earliest spike were subjectively selected and the average fluorescence intensity (F) in these images was set as the basal level. Changes in fluorescence intensity (ΔF) in the images were calculated and ΔF/F was used to denote Ca^2+^ responses. Pseudocolour images were generated with Matlab (Mathworks Inc., Natick, MA, USA) by setting the basal fluorescence level at zero.

### Immunohistology and fluorescence quantification

We generated an antibody to Dilp2 by immunizing rabbits with the *Drosophila* Dilp2 peptide sequence TRQRQGIVERC (ref. 9). Brains were dissected from larvae in PBS, fixed in PBS containing 4% formaldehyde for 60 min at room temperature and washed four times for 30 min each in PBS containing 0.5% Triton X-100 (PBT) before blocking for 1 h in PBT containing 10% goat serum. The samples were then incubated with primary antibodies (rabbit anti-Dilp2, 1:1,000 or rabbit anti-CD4, 1:200, Epitomics Inc., Burlingame, CA, USA) overnight at 4 °C, followed by four 30 min washes in PBT. The samples were then incubated with secondary antibody (TRITC-conjugated goat anti-rabbit, 1:100, Molecular Probes Inc., Grand Island, NY, USA) for 2 h at room temperature. Tissues were mounted and viewed. Images were acquired using an Olympus FV1000 confocal laser scanning microscope.

To quantify Dilp2 or GFP levels in CaLexA imaging, confocal Z series of the cell bodies of IPCs or axonal termini of *11216-Gal4* neurons were acquired at a step size of 1 μm. The same laser power and scanning settings were used for all samples. ImageJ was used to generate a sum-intensity Z stack projection and measure total fluorescence intensity.

### Confocal microscopy

To compare the expression patterns of *11216-Gal4* and Or83b-RFP in larval head and brain, *11216-Gal4* was crossed with *Or83b-RFP*;*UAS-mCD8-GFP* and expression patterns were checked in progeny larvae. For expression patterns in larval head, heads of early third-instar larvae were removed from the body and incubated in 50% glycerol in PBS for 1 h before being viewed^33^. For expression patterns in larval brain, middle third-instar larval brains were dissected and fixed in PBS containing 4% formaldehyde for 60 min at room temperature before being washed four times for 30 min each in PBS. Brain tissues were then mounted and viewed. For both head and brains, images were acquired using an Olympus FV1000 confocal laser scanning microscope and processed with ImageJ.

## Additional information

**How to cite this article:** Li, Q. *et al*. Cold-sensing regulates *Drosophila* growth through insulin-producing cells. *Nat. Commun.* 6:10083 doi: 10.1038/ncomms10083 (2015).

## Supplementary Material

Supplementary Information Supplementary Figures 1-21 and Supplementary Table 1

Supplementary Movie 1 Ca^2+^ imaging of IPCs in response to cold. Real-time movie shows the GCAMP6.0 signal in two clusters of cell bodies of IPCs in an early 3^rd^-instar *dilp2-Gal4/+;UAS-GCAMP6.0/+* larva in response to a temperature decrease as shown in Figure 2a,b. Temperature is indicated in the caption.

Supplementary Movie 2 Ca^2+^ imaging of IPCs in response to temperature rise. Real-time movie shows the GCAMP6.0 signal in two clusters of cell bodies of IPCs in an early 3^rd^-instar *dilp2-Gal4/+;UAS-GCAMP6.0/+* larva in response to a temperature rise from 25.0°C to 30.5°C as plotted in Supplementary Figure 5. Temperature is indicated in the caption.

Supplementary Movie 3 Ca^2+^ imaging of 11216-Gal4 neurons in response to a temperature drop. Real-time movie shows the GCAMP6.0 signal in clusters of neuronal cell bodies in the rostral tip of an early 3^rd^-instar *11216-Gal4/+;UAS-GCAMP6.0/+* larva in response to a temperature decrease from 25°C to 18°C and restoration as shown in Figure 3d,e. Temperature is indicated in the caption.

Supplementary Movie 4 Ca^2+^ imaging of *11216-Gal4* neurons in response to a temperature rise. Real-time movie shows the GCAMP6.0 signal in clusters of neuronal cell bodies in the rostral tip of an early 3^rd^-instar *11216-Gal4/+;UAS-GCAMP6.0/+* larva in response to a temperature rise from 25.0°C to 30.2°C as plotted in Supplementary Figure 12. Temperature is indicated in the caption.

Supplementary Movie 5 Ca^2+^ imaging of IPCs in response to optogenetic activation of *11216-Gal4* neurons. Ca^2+^ response of IPCs was seen when *11216-Gal4* neurons expressing Chrimson were stimulated by red light for 50 sec as shown in Figure 4e. “red light” indicates the application of stimulation.

Supplementary Movie 6 Ca^2+^ response of IPCs in a larva expressing GCAMP6.0 in IPCs but without Chrimson expression in *11216-Gal4* neurons. A 50 sec exposure of red light was used as optogenetic stimulation as shown in Supplementary Figure 18. “red light” indicates the application of stimulation.
