# Supplementary material for: Cold-sensing regulates Drosophila growth through insulin-producing cells
Source: Nat Commun. 2015 Dec 9;6:10083. doi: 10.1038/ncomms10083 (PMC4682036; doi:10.1038/ncomms10083)
Supplement: Supplementary Information — Supplementary Figures 1-21 and Supplementary Table 1 [file ncomms10083-s1.pdf]

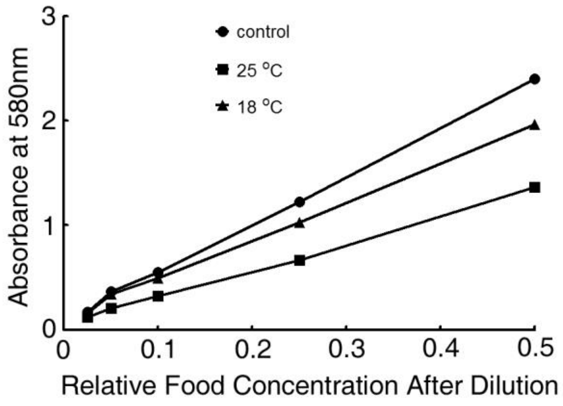

Supplementary Figure 1 | 580 nm absorbance of iodo-starch reactions using different dilutions of food from three samples. The three samples were: food prior to culture and food after flies were cultured at the two indicated temperatures. The food concentrations after serial dilutions were relative to original food solution. See Methods for details.

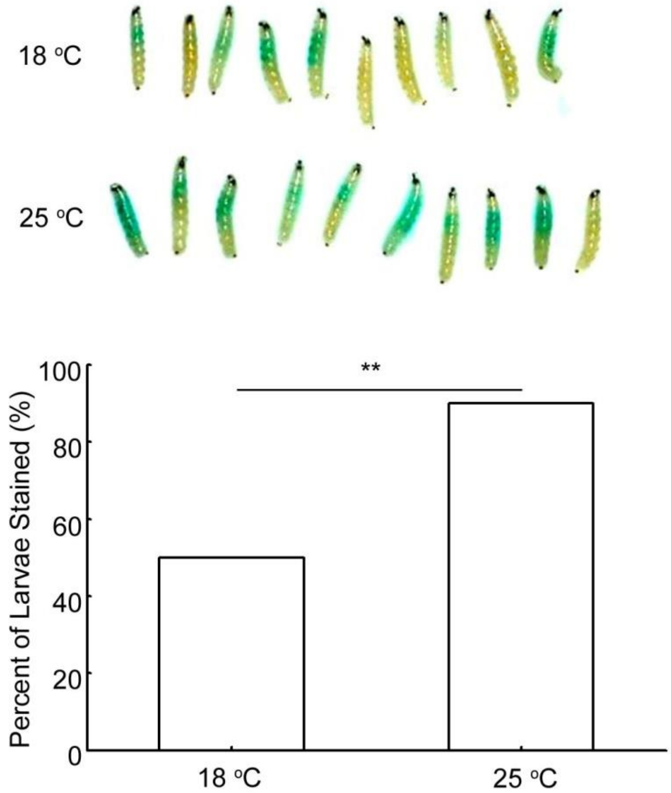

Supplementary Figure 2 |*w*^{1118} larvae ate more slowly at 18°C than at 25°C. (**a**) Images of *w*^{1118} larvae after 20 minutes consuming dye-containing food at 18°C and 25°C. (**b**) Statistics of **a**. n = 10 for both conditions. ***P*<0.01, Mann-Whitney test.

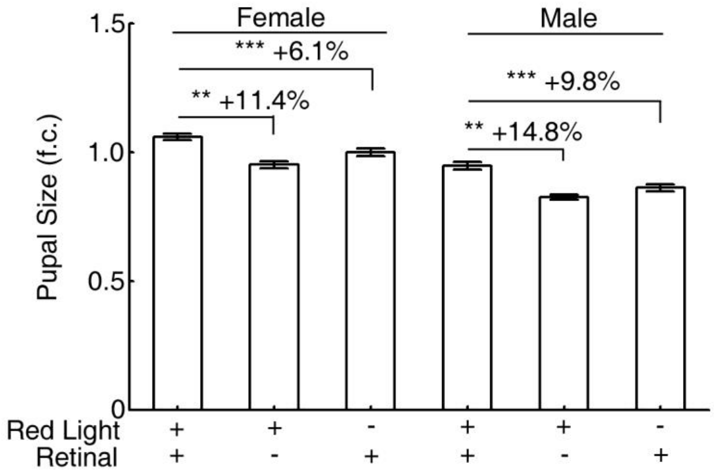

Supplementary Figure 3 |Optogenetically activating IPCs enhanced pupal size in both females and males. Flies expressing *UAS-Chrimson* with *dilp2-Gal4* were cultured under 620-nm red light on food supplied with all-trans-retinal to stimulate IPCs (n = 14 for females; n = 10 for males). Error bars are s.e.m., ***P*<0.01, ****P*<0.001, student's *t*-test.

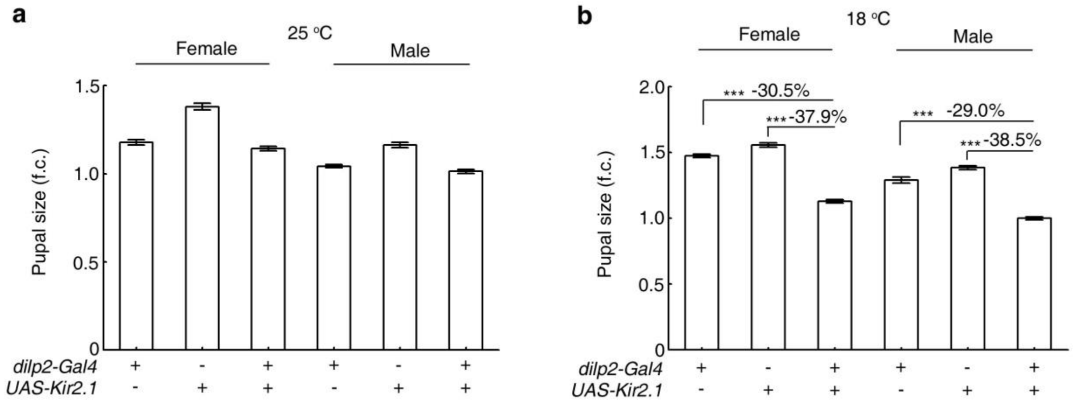

Supplementary Figure 4 |Pupal sizes of flies with IPCselectrically-silenced. Pupal sizes of flies with IPCs silenced by Kir2.1 were decreased when cultured at 18°C (a) but not at 25°C (b). n = 13 for all. Error bars are s.e.m., ****P*<0.001, student's *t*-test.

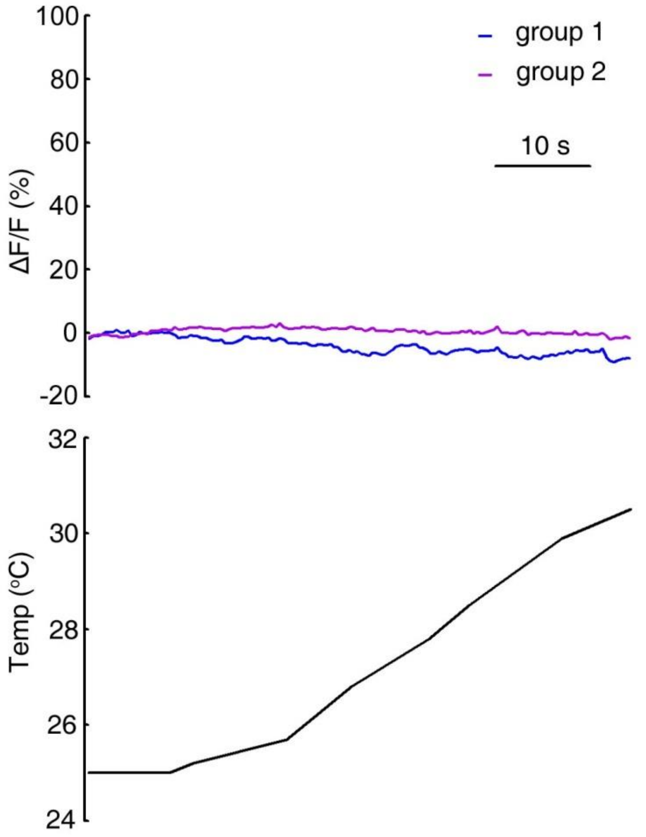

Supplementary Figure 5 |Ca^{2+} imaging of larval IPCs in response to a temperature rise from 25.0°C to 30.5 °C. No significant response was observed.

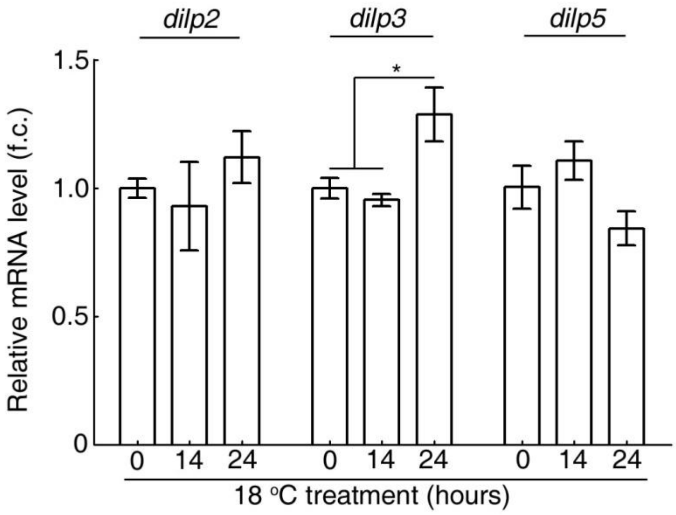

Supplementary Figure 6 |Brain *dilp2, dilp3, and dilp5* mRNA expression levels after 14 and 24 h culture at 18°C (n = 3). Error bars are s.e.m., *P<0.05, student's *t*-test.

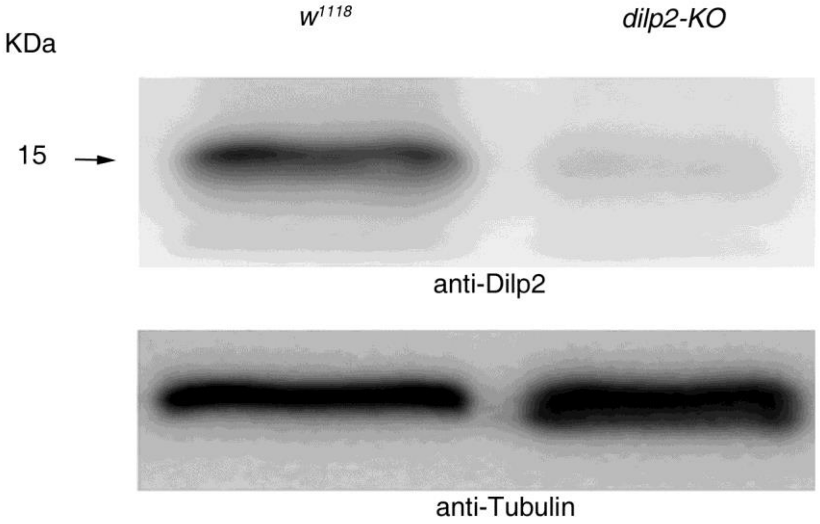

Supplementary Figure 7 | Verification of anti-Dilp2 antibody in western blots using *dilp2-KO* flies. The 15K Da anti-Dilp2 band was strong in w^{1118} but barely detectable in *dilp2-KO*.

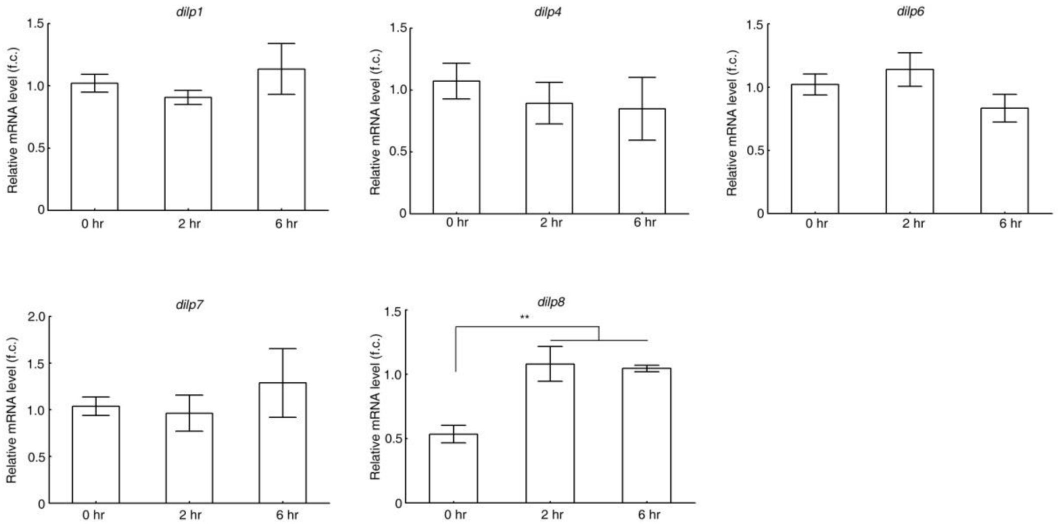

Supplementary Figure 8 | Whole larval body *dilps1-8* mRNA expression levels after 0, 2 and 6 h culture at 18°C. Except for *dilp8*, whose expression was doubled, no significant changes were observed for all *dilps* (n = 3). Error bars are s.e.m., $^{**}P<0.01$, student's *t*-test.

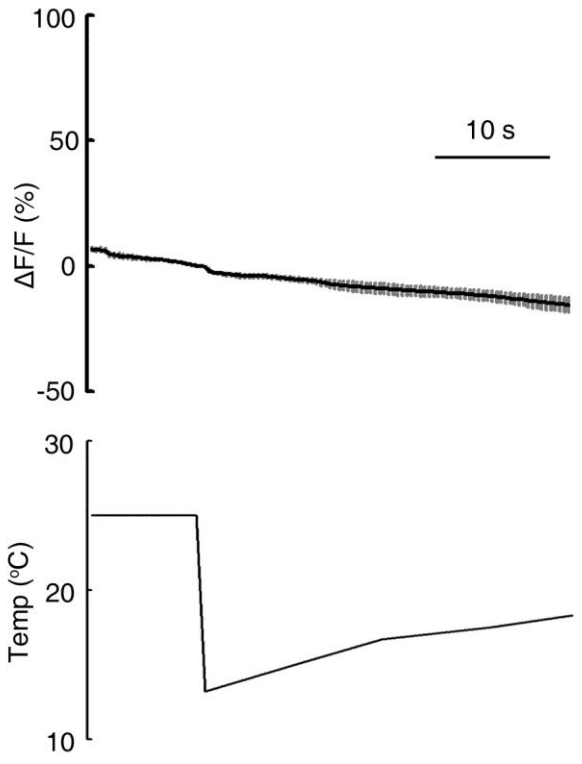

Supplementary Figure 9 | Ca^{2+} imaging of fat body cells in response to a rapid temperature drop. Expression of *UAS-GCAMP6.0* in fat bodies was driven by *cg-Gal4*. No significant response was observed (n = 3).

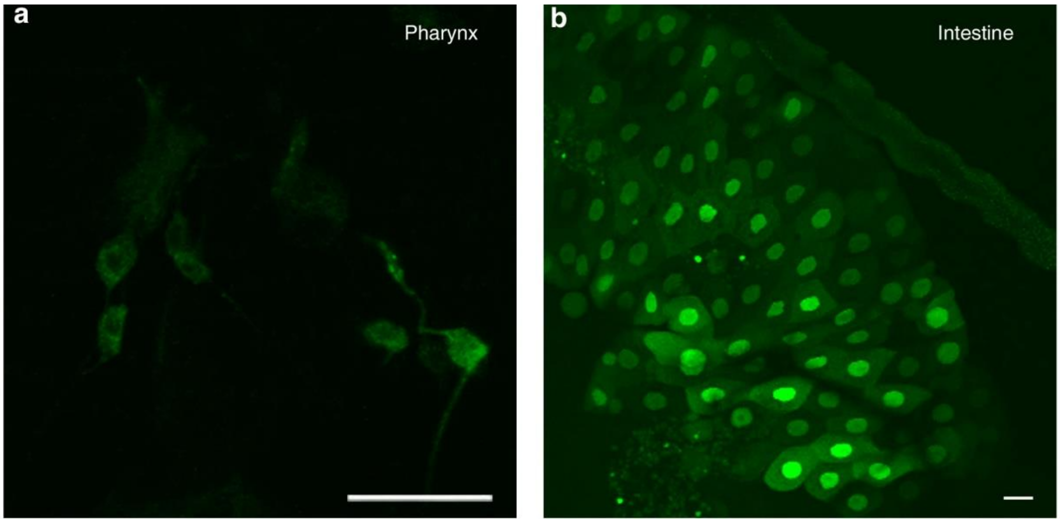

Supplementary Figure 10 |Expression of *11216-Gal4* in larval pharynx and intestinal cells. (**a**) *UAS-mCD8-GFP* was used to label cells in the pharynx. (**b**) *UAS-nls-GFP* was used to label nuclei of intestinal cells. Scale bars denote 50 µm.

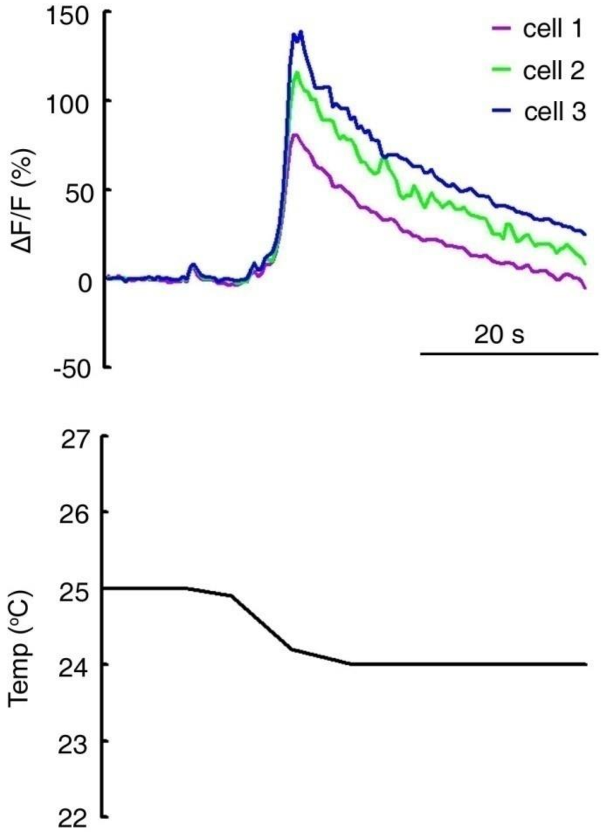

Supplementary Figure 11 |Ca^{2+} imaging of *11216-Gal4*-labelled neurons in response to a temperature decrease from 25°C to 24°C. Imaging of three *11216-Gal4* neurons in one representative sample is shown as fluorescence intensity curves. A strong response occurred when the temperature decrease just began, while still much less than 1°C.

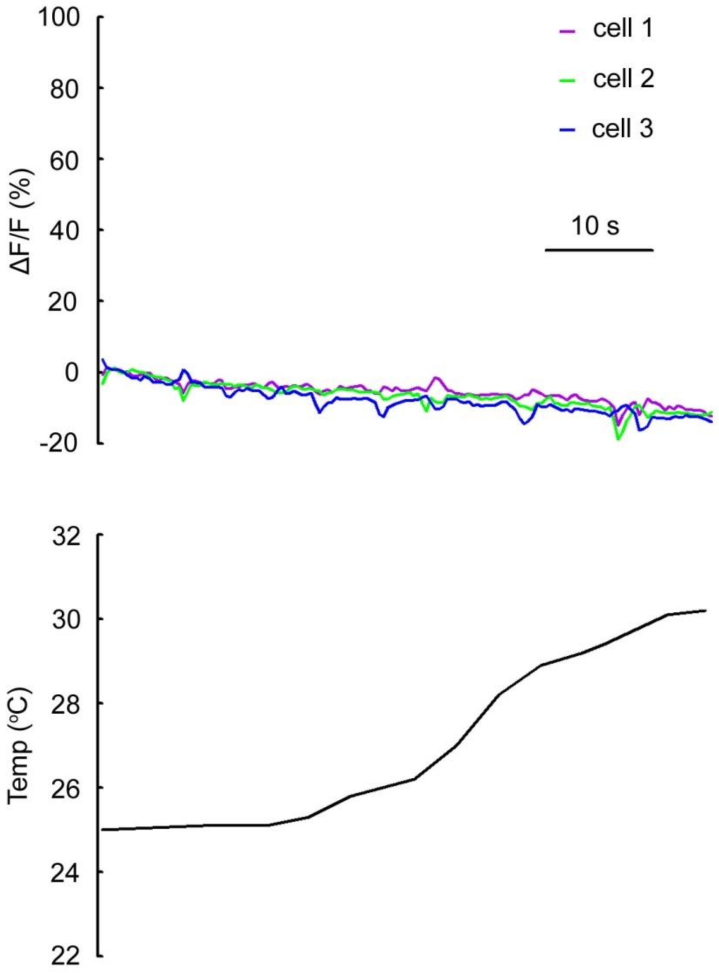

Supplementary Figure 12 |Ca^{2+} **imaging of larval *11216-Gal4* neurons in response to a temperature rise from 25.0°C to 30.2°C in a representative sample.**

No significant response was observed in three representative neurons.

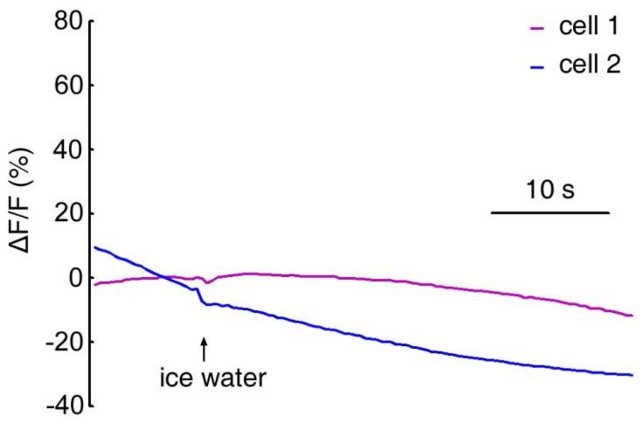

Supplementary Figure 13 |Ca^{2+} imaging of *11216-Gal4*-labelled larval intestinal cells in response to a rapid temperature drop in a representative sample. No significant response was observed in two representative cells.

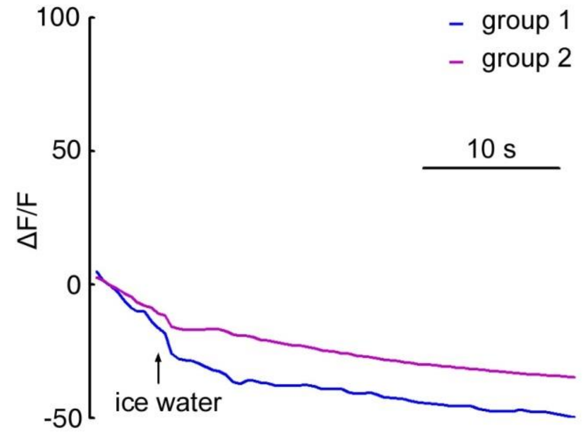

Supplementary Figure 14 |Ca^{2+} imaging of *11216-Gal4*-labelled larval pharyngeal neurons in response to a rapid temperature drop in a representative sample. No significant response was observed in two representative groups of cells.

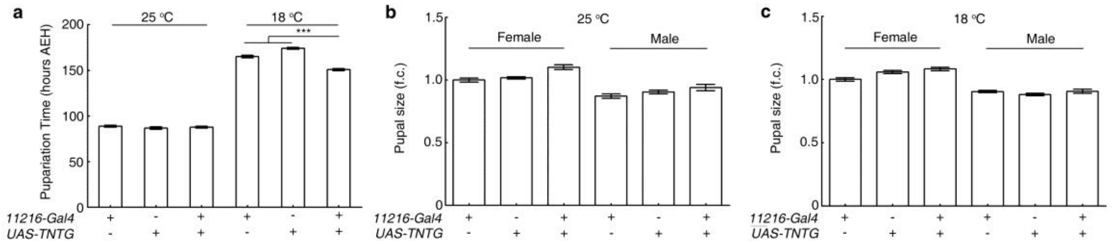

Supplementary Figure 15 |Blocking *11216-Gal4* neurons with *UAS-TNTG* was not sufficient to reverse the effects of cold temperature on pupal size and pupariation. (**a**) Pupariation time was earlier in flies with *11216-Gal4* neurons blocked by *UAS-TNTG* at 18°C but not at 25°C, as compared with the controls (n = 8). (**b,c**) At both 25°C (**b**) and 18°C (**c**) pupal sizes of flies with *11216-Gal4* neurons blocked by *UAS-TNTG* were not reduced as compared with those of controls (n = 17). Error bars are s.e.m., ***P<0.001, student's t-test.

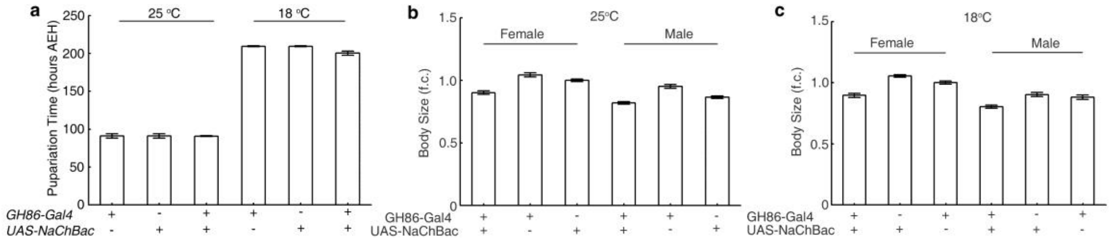

Supplementary Figure 16 |Activating *GH86-Gal4* neurons with *UAS-NaChBac* did not affect pupal size and pupariation. (**a**) Pupariation time was not affected in flies with *GH86-Gal4* neurons activated by *UAS-NaChBac* as compared with the controls, at both 25°C and 18°C (n = 8). (**b,c**) At both 25°C (**b**) and 18°C (**c**), pupal sizes of flies with *GH86-Gal4* neurons activated by *UAS-NaChBac* were not different from those of controls (n = 13). Error bars are s.e.m., student's t-test was used.

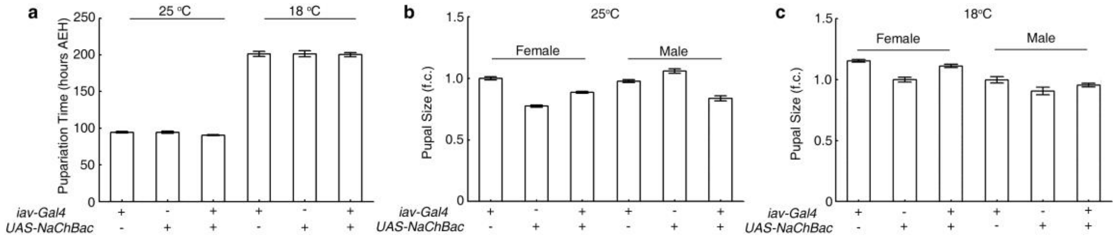

Supplementary Figure 17 |Activating *iav-Gal4* neurons with *UAS-NaChBac* did not affect pupal size and pupariation. (**a**) Pupariation time was not affected in flies with *iav-Gal4* neurons activated by *UAS-NaChBac* as compared with in the controls, at both 25°C and 18°C (n = 6). (**b,c**) At both 25°C (**b**) and 18°C (**c**), pupal sizes of flies with *iav-Gal4* neurons activated by *UAS-NaChBac* were not different from those of controls (n = 17). Error bars are s.e.m., student's *t*-test was used.

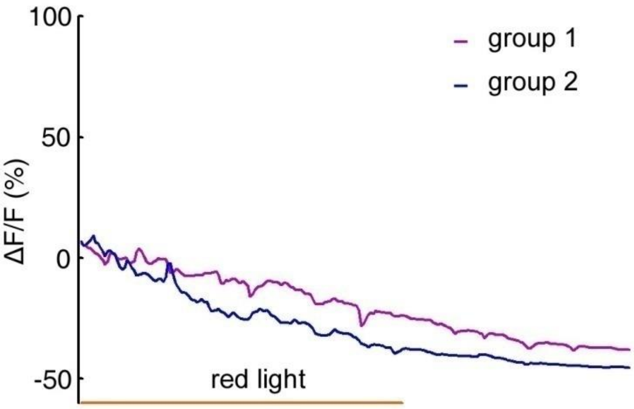

Supplementary Figure 18 |Fluorescence intensity of two groups of larval IPC signals in a representative *dilp2-LexA;LexAop-GCAMP6.0* sample upon red-light stimulation. No significant response was observed.

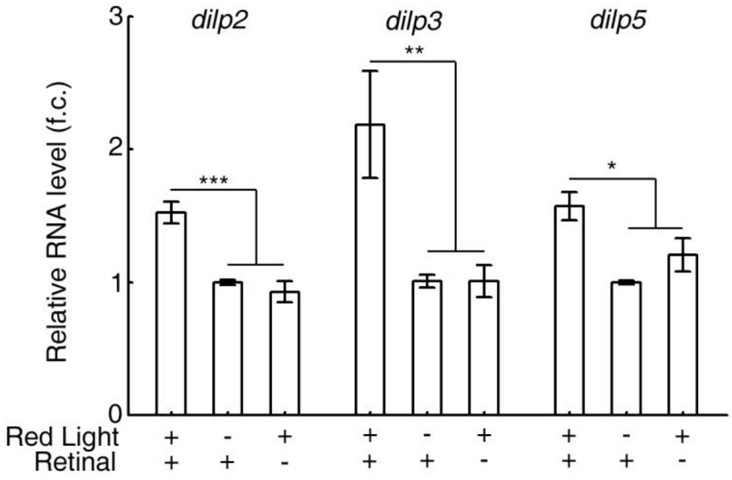

Supplementary Figure 19 |Larvae with *11216-Gal4* neurons optogenetically activated showed higher mRNA levels of *dilp2, dilp3 and dilp5*. Flies expressing *UAS-Chrimson* with *11216-Gal4* were cultured under 620-nm red light on food supplied with all-trans-retinal to stimulate *11216-Gal4* neurons (n = 3). Error bars are s.e.m., *P<0.05, **P<0.01, ***P<0.001, student's t-test.

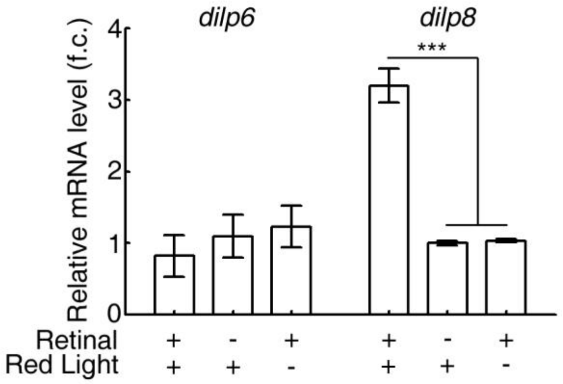

Supplementary Figure 20 |Optogenetically activating *11216-Gal4* neurons enhanced mRNA expression of *dilp8* but not of *dilp6* in whole larval bodies. Flies expressing *UAS-Chrimson* with *11216-Gal4* were cultured under 620-nm red light on food supplied with all-trans-retinal to stimulate IPCs (n = 3). Error bars are s.e.m., ***P<0.001, student's t-test.

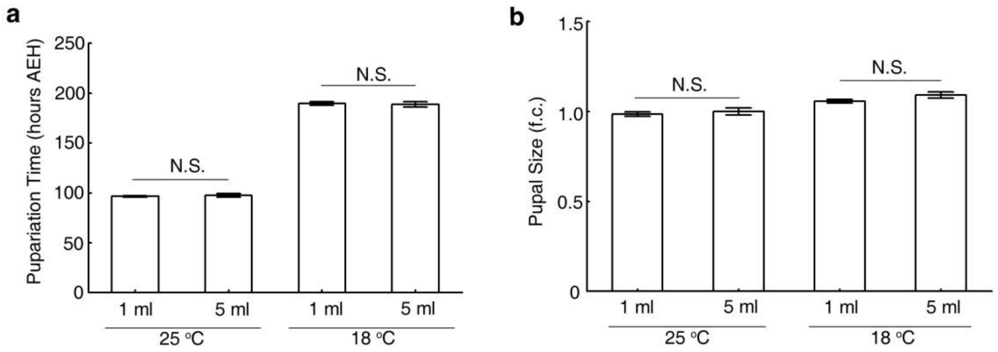

Supplementary Figure 21 | Pupal size and pupariation time of *w*^{1118} were not affected by two different culturing densities. Two culturing densities were used: 20 larvae on 1ml food (indicated as 1ml) and 40 larvae on 5ml food (indicated as 5ml). (**a**) Pupariation time of *w*^{1118} larvae raised at two densities was not different at both 18 °C and 25°C. n = 10 for all groups. (**b**) Pupal sizes of *w*^{1118} larvae raised at two densities were not different at both 18 °C and 25°C. Both pupariation time and pupal size were measured *en masse* for females and males. n = 16 for all groups. Error bars are s.e.m., N.S., statistically not significant, *P*>0.05, student's *t*-test.

Supplementary Table 1.Expression of 11216-Gal4 in larval tissues

tissue	expression
Anterior terminal	Yes
Pharynx	Yes
CNS	Yes
PNS	No
Salivary gland	No
Gut	Yes
Skin	No
Muscle	No
